# Racemose Neurocysticercosis

**DOI:** 10.4269/ajtmh.19-0868

**Published:** 2020-04

**Authors:** Susan Y. Yang, William W. Lines, Carlos M. Vásquez, for the Cysticercosis Working Group in Perú

**Affiliations:** 1 School of Medicine, Trinity College Dublin, Dublin, Ireland;; 2 Unidad de Investigación de Enfermedades Parasitarias del Sistema Nervioso, Universidad Peruana Cayetano Heredia, Lima, Perú;; 3 Centro Básico de Investigación en Exámenes Auxiliares en Parasitosis del Sistema Nervioso, Instituto Nacional de Ciencias Neurológicas, Lima, Perú;; 4 Department of Neurosurgery, Instituto Nacional de Ciencias Neurológicas, Lima, Perú

A 53-year-old woman presented to the emergency department of the National Institute of Neurological Sciences in Lima, Peru, with a 3-month history of severe headache, confusion, and incoherent speech. Neurological examination showed preserved strength in all limbs, preserved coordination, and normal oculomotor function. Serum Western immunoblot for cysticercosis was strongly positive. Circulating antigens for *Taenia solium* were positive with saturating levels (antigen optical density ratio > 70.0). Magnetic resonance imaging of the head revealed multiple conglomerate cystic lesions of different sizes in the basal cisterns surrounding the brainstem, the anterior interhemispheric cistern, and the Sylvian cisterns (Figure 1).

**Figure 1.**
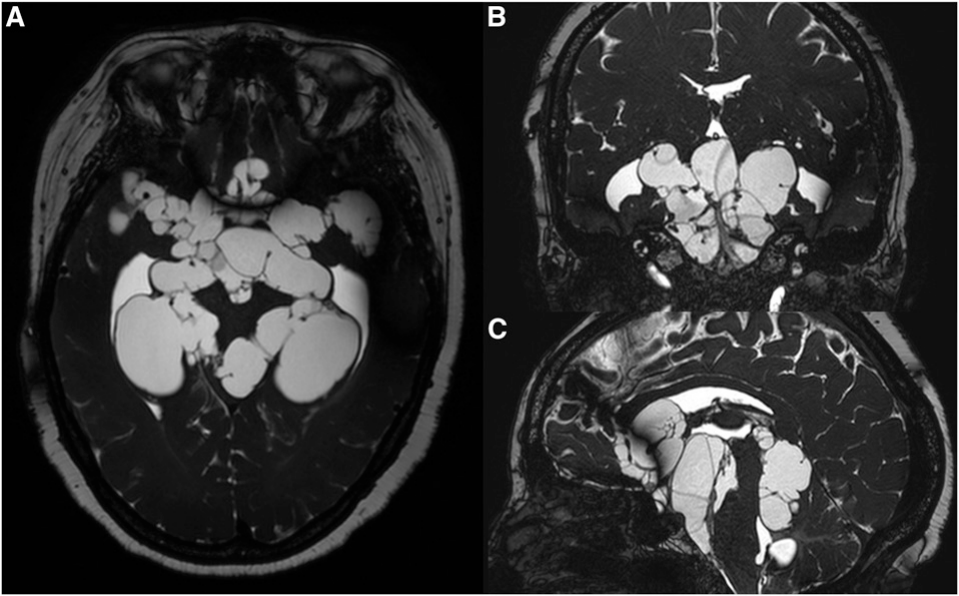
Magnetic resonance imaging of the head with gadolinium contrast in fast imaging employing steady-state acquisition sequence showing extent of cystic lesions in the basal cisterns in (**A**) axial, (**B**) coronal, and (**C**) sagittal sections.

Two weeks later, the patient underwent endoscopic transnasal resection of cysticerci (Figures 2 and 3A). Pathological examination of the parasite showed cysticerci with tegumental membranes typical of *T. solium* and no scolex (Figure 3B).

**Figure 2.**
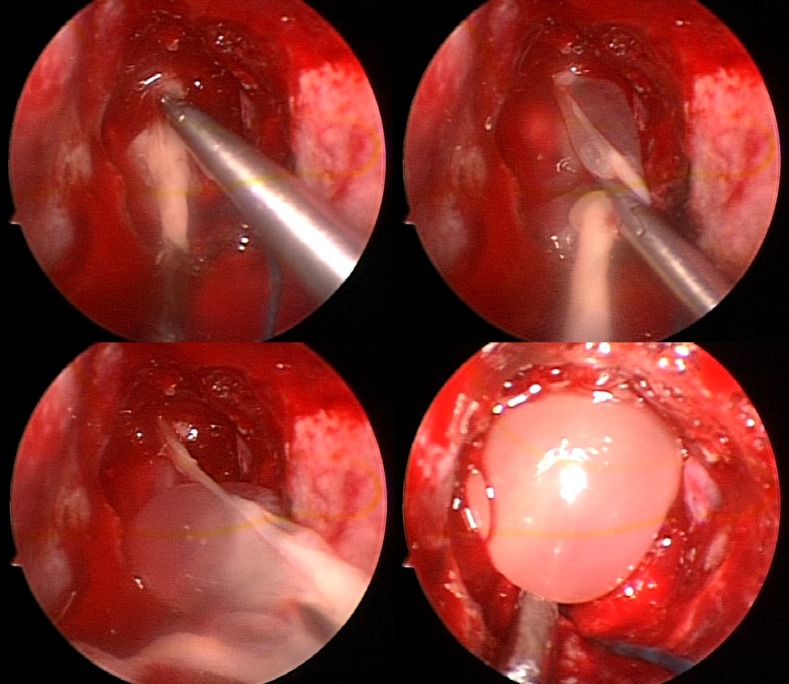
Endoscopic transnasal resection of racemose cysticerci. This figure appears in color at www.ajtmh.org.

**Figure 3.**
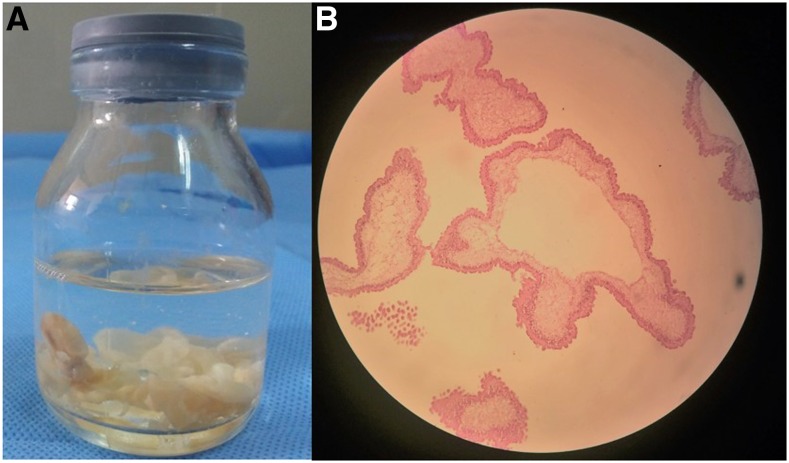
(**A**) Extracted cysticerci membranes. (**B**) Microscopic pathologic image demonstrating a cysticercus with tegumental membrane typical of *Taenia solium*. Hematoxylin and eosin stain. Scolex is not visualized. This figure appears in color at www.ajtmh.org.

At 6-week follow-up, the patient no longer had any symptomatic complaints and was put on phenytoin, along with a course of albendazole and dexamethasone. In patients with intraparenchymal cysticerci, praziquantel combined with albendazole has been shown to be the most efficacious treatment regimen in achieving cyst resolution at 6-months follow-up.^1^ Praziquantel was not co-administered to this patient in part because of restraints in its cost and availability in Peru. In addition, experience in using the praziquantel–albendazole combination therapy for subarachnoid neurocysticercosis (NCC) is limited, although there is currently an ongoing randomized trial being conducted by the Cysticercosis Working Group in Perú.

At last follow-up at 2 months postoperative, the patient presented in good general condition with slight dizziness and complained of mild intermittent headaches which she attributed to stress. She scored 15 on the Glasgow Coma Scale and had no focal neurological deficits. The patient’s recovery is ongoing and is followed regularly by a neurologist and neurosurgeon.

Neurocysticercosis is a parasitic infection of the central nervous system by the larval form of the pork tapeworm *T. solium*, with the most common symptoms being headache and seizure. Racemose NCC is a form of the disease characterized by multiple confluent cysts in the subarachnoid space and is associated with hydrocephalus, intracranial hypertension, vasculitis, and cerebrovascular events. In cases of racemose NCC, the scolex is characteristically absent and rarely visualized in the fluid-filled bladder of the cyst (Figures 2 and 3).^2^ The usual management of subarachnoid NCC involves long courses of antiparasitic drugs with concomitant corticosteroids. However, if massive amounts of cysts are present, as in this case, antiparasitic treatment alone is usually not sufficient and surgical intervention is required.^2,3^

## Supplemental figure

Supplemental materials
