# Supplementary material for: Racemose Neurocysticercosis
Source: Am J Trop Med Hyg. 2020 Apr;102(4):703–4. doi: 10.4269/ajtmh.19-0868 (PMC7124892; doi:10.4269/ajtmh.19-0868)

supplemental figure 1:

Computed tomography imaging at seven weeks post-operative showing a reduced number of cystic lesions in the basal cisterns.

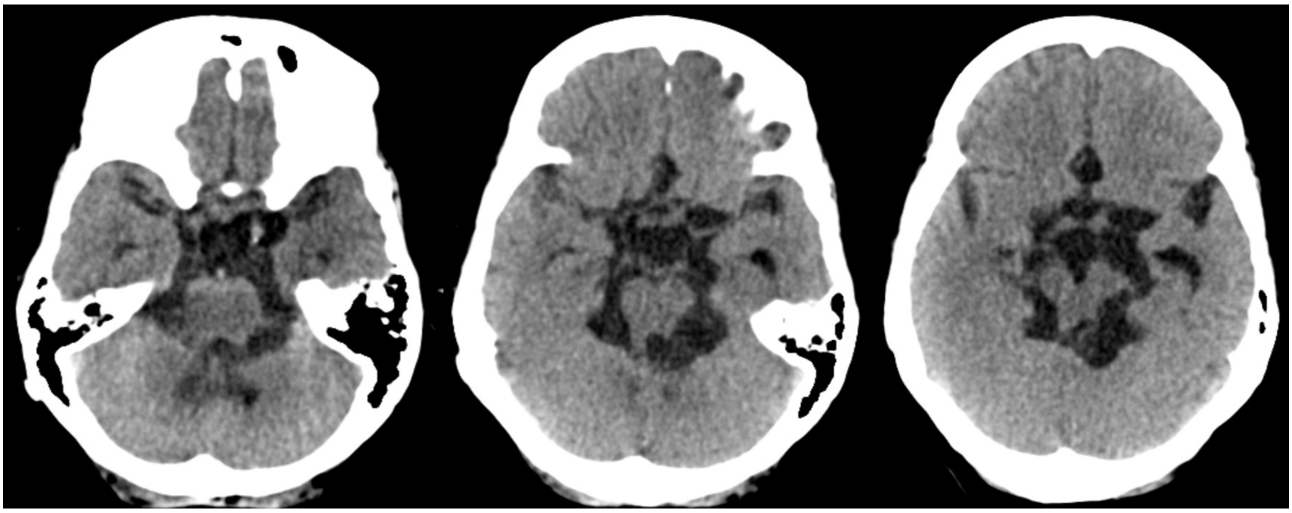

Supplement: Supplementary file 1 [file tpmd190868.SD1.pdf]
